# Motor cortex in levodopa-induced dyskinesia: Systems and molecular changes after sub-anesthetic ketamine treatment

**DOI:** 10.4103/NRR.NRR-D-25-00176

**Published:** 2025-09-29

**Authors:** Torsten Falk, Stephen L. Cowen

**Affiliations:** Psychology Department, The University of Arizona, Tucson, AZ, USA; Department of Neurology and Department of Pharmacology, The University of Arizona, Tucson, AZ, USA

The loss of control over movement is one of the most devastating consequences of Parkinson’s disease (PD). The loss of control largely results from the gradual but inexorable destruction of dopamine-producing neurons in the *substantia nigra pars compacta*. As dopamine levels fall, the ability to initiate, control, learn, and sustain actions declines. Treatment with the dopamine precursor levodopa can partly overcome motor impairments; however, years of use often leads to levodopa-induced dyskinesia (LID), a debilitating condition characterized by uncontrolled writhing and ballistic movements, making continued treatment difficult or impossible. While progress has been made towards unraveling the molecular and cellular processes driving the development of LID, far less is known about the changes in ongoing neuronal activity that contribute to LID expression.

While LID involves many interconnected cortical and subcortical systems, the primary motor cortex (M1) is often implicated. What follows is a review and perspective of the role M1 single-unit and neural ensemble activity play in LID expression and how low-dose ketamine may exert anti-dyskinetic effects by reconfiguring between-neuron interactions.

**LID decouples primary motor cortex activity from ongoing movements:** Understanding the role of M1 in LID requires an understanding of the role of M1 in the normal control of movement. The original work of Penfield and Boldrey (1937) indicated that M1 is organized as a “motor homunculus,” where localized groups of neurons form a one-to-one somatotopic map with downstream spinal neurons and associated muscle groups. This view has largely shifted in favor of a population-based interpretation. While M1 does indeed express a coarse topography, movements, and motor plans appear to be controlled by the weighted activities of populations of neurons distributed throughout the motor cortex. This ensemble-level perspective is supported by anterograde and retrograde tracing studies indicating that M1 neurons connect to a diverse set of downstream regions, as well as by the original work of Georgopoulos et al. (1986) demonstrating that arm movements can be faithfully predicted by the weighted activities of ensembles of neurons.

Considerable evidence indicates that M1 neurons excite and activate downstream motor circuits. Less appreciated is the capacity of M1 neurons to inhibit motor centers and suppress unwanted movements. Indeed, Penfield noted that, in some patients, electrical stimulation suppressed movement and relaxed muscles. Furthermore, M1 principal neurons project to D2-receptor-expressing medium spiny neurons in the indirect pathway, a pathway implicated in action inhibition and selection. In addition, M1 layer 5 principal neurons project to inhibitory neurons in the brain stem and spinal cord, and physiological data indicate that motor cortex neurons inhibit spinal motor neurons (Jankowska et al., 1976). Taken together, these data suggest motor cortex involvement in both suppressing competing and activating planned actions.

Theories of LID generally take a “motor activation” view of M1, suggesting that aberrant M1 activity triggers unwanted movement. This implies that M1 neural activity should be tightly coupled to ongoing dyskinetic movements. How motor inhibition relates to LID is less clear. It is conceivable that LID disrupts the capacity of M1 to properly suppress unwanted movements, resulting in their spontaneous emergence from dysregulated downstream motor circuits. If M1 motor inhibition is a major component of LID, it is less likely that changing M1 activity would be correlated with dyskinetic movement as this activity would relate to the suppressed movements and not those that ultimately emerge from downstream generators.

Recently published data from our group cast doubt on the motor-activation view of M1 involvement in LID (Vishwanath et al., 2024). In this study, local-field potentials and single-unit activity from > 3000 M1 neurons were acquired using the rat 6-OHDA model of PD and LID. Precise measurement of movement was measured using head-mounted inertial sensors, allowing the investigation of neurophysiological and motor signatures of LID with high temporal precision.

Movement in healthy animals is associated with increased wide-band gamma (35–120 Hz) in M1. In contrast, a focal 80 Hz rhythm, called finely-tuned gamma, is a signature of LID. In our experiment, we observed that moment-to-moment fluctuations in M1 wide-band gamma and single-unit firing were positively correlated with spontaneous movements in sham and PD animals. In accordance with previous findings, we also found that lower-frequency beta-band activity (15–30 Hz) was negatively correlated to movement. These relationships changed significantly during dyskinesia. When levodopa was administered to the LID animals, correlations between local-field and single-unit activity and movement approached zero, suggesting that M1 becomes functionally decoupled from movement-initiating circuits during LID. This puts into question the idea that M1 neurons directly trigger dyskinetic actions. Instead, these data suggest, albeit indirectly, that in LID M1 loses its capacity to properly constrain downstream motor circuits, freeing them to spontaneously generate involuntary movements. This is reasonable as downstream circuits in the brainstem are capable of independently generating orofacial, locomotor, and other dyskinetic movements associated with LID. Thus, it is conceivable that the role of M1 in LID results from a failure to properly inhibit unwanted movement rather than triggering them. This, however, is probably not due to an absence of M1 signals reaching downstream motor circuits as M1 activity is increased in LID (Halje et al., 2012). This activity, however, may be dysregulated, resulting in the disorganized suppression of downstream circuits. Disordered motor inhibition would help explain why, in our study, M1 single-unit activity was so notably uncorrelated with movement.

**Ketamine reduces LID and disrupts neural synchronization associated with LID:** At high doses, ketamine is an effective general anesthetic. At lower doses, ketamine can have fast-acting and weeks-long anti-depressant effects and effectively treat chronic pain and migraine headaches. Our group has found evidence from animal studies indicating that sub-anesthetic ketamine reduces LID for weeks following a single extended administration (Bartlett et al., 2016) and attenuates LID when given during levodopa priming (Bartlett et al., 2020), suggesting that ketamine delays LID onset and limits severity, and may show acute anti-parkinsonian activity. Little is known regarding how ketamine alters local-field or neural-ensemble activity in health or disease. Consequently, we investigated how ketamine affects neural synchrony and neuron-neuron interactions in the experiment described above.

In this experiment, M1 neural activity was measured in sham, PD, and dyskinetic animals following dopamine precursor levodopa and ketamine administration. We found that a single sub-anesthetic dose of ketamine produced a near immediate reduction in LID and the elimination of LID-associated 80-Hz finely-tuned gamma. Importantly, ketamine enhanced the correlation between ongoing M1 activity and movement, indicating that ketamine partially re-established M1 regulation of downstream motor centers, suggesting a potential mechanism by which ketamine exerts its anti-dyskinetic effects.

While indicative, this observation does not speak directly to how ketamine alters coordinated neuronal activity or how it partially restores the connection between M1 and movement. In the previously described experiment, neural ensemble activity from up to 50 simultaneously recorded neurons was acquired from M1 using tetrodes. This allowed us to quantify between-neuron interactions during control, ketamine-alone, and ketamine + LID (ketamine delivered to LID animals that received levodopa) conditions. Under each condition, a matrix of all possible between-neuron correlations was generated, where each cell in the correlation matrix indicated the degree ongoing activities of a given neuron pair were correlated, anti-correlated, or uncorrelated.

**Figure 1:**
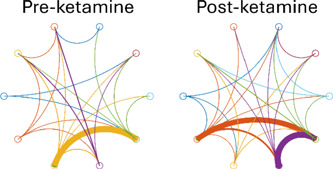
Ketamine-induced rearrangement of cell-pair correlations in levodopa-induced dyskinesia. Each node (circle) indicates an individual neuron. Line width indicates the strength of correlated activity. Colors indicate the identity of one of the neuron pairs. The plot illustrates the capacity of ketamine to re-organize the pattern of correlations between neurons. This effect is particularly strong in levodopa-induced dyskinesia.

We observed that ketamine profoundly reorganized and, in a sense, “shuffled” the pattern of M1 neuron-pair correlations. Specifically, many neuron-pairs that were formerly correlated prior to ketamine became uncorrelated or negatively correlated following ketamine administration (**Figure 1**). The reverse was also true where formerly uncorrelated or negatively correlated cell-pairs changed, and this change was greatest when ketamine was given during LID.

This network-wide change in activity may result from ketamine-mediated modulation of M1 parvalbumin-expressing (PV) neurons. PV neurons exert powerful somatic inhibition of M1 principal cells. Ketamine has a high affinity and is an antagonist for N-methyl-D-aspartate receptors (NMDARs) with the GLuN2B subunit, and this form of NMDAR is particularly abundant in PV neurons (Lewis et al., 2022). Thus, ketamine-mediated modulation of even a small population of PV neurons could radically reshape network activity. While altered cell-pair correlations and the partial re-coupling of M1 activity to movement may underlie the immediate anti-dyskinetic effects of ketamine, these effects do not explain how or whether such changes contribute to weeks-long effects of ketamine in LID. The following section explores how ketamine may induce lasting changes in motor circuits.

**Possible cellular and molecular mechanisms of action:** While ketamine itself is metabolized within hours, it triggers the release of brain-derived neurotrophic factor (BDNF), as reviewed by Zanos and Gould (2018), and other factors capable of inducing lasting neuroplastic change. Indeed, the long-term anti-dyskinetic activity of ketamine was recently found to be dependent on BDNF-signaling and can be completely blocked by a tropomyosin receptor kinase B (TrkB)-antagonist (Bartlett et al., 2020). This mirrors what has been shown in models of depression, where NMDAR antagonism is at the core of the *cortical disinhibition hypothesis* for the acute anti-depressive activity of ketamine, and ketamine-induced BDNF-signaling is thought to underly long-term anti-depressive effects (Zanos and Gould, 2018).

**Figure 2:**
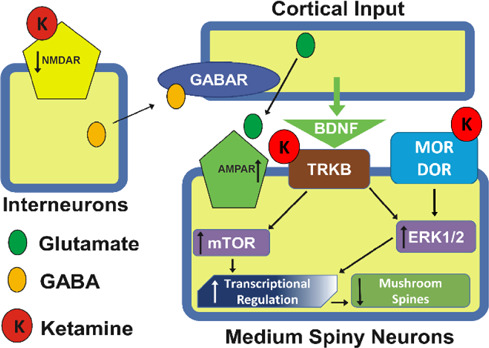
Cortical disinhibition hypothesis. Ketamine (K) causes a burst of glutamate due to reduced cortical GABA PV interneuron activity in M1; leading to glutamate and BDNF release onto downstream striatal MSNs, activating AMPARs, depolarization, and to stimulation of TrkB, activating mTORC1 and ERK1/2. K also binds and stabilizes TrkB, enhancing BDNF signaling further, and leading to increased protein synthesis required for synaptogenesis and plasticity. In addition, K is a PAM for MORs/DORs, known to activate ERK1/2, leading to acute anti-parkinsonian effects. AMPARs: α-Amino-3-hydroxy-5-methyl-4-isoxazolepropionic acid receptors; BDNF: brain-derived neurotrophic factor; DOR: delta-opioid receptor; ERK1/2: extracellular signal-regulated kinase 1/2; GABA: gamma-aminobutyric acid; GABAR: gamma-aminobutyric acid receptor; K: ketamine; M1: motor cortex; MOR: mu-opioid receptor; MSNs: medium spiny neurons; mTOR: mammalian target of rapamycin; mTORC1: mammalian target of rapamycin complex 1; NMDAR: N-methyl-D-aspartate receptor; PAM: positive allosteric modulator; PV: parvalbumin; TRKB: tropomyosin receptor kinase B.

At the molecular level (**Figure 2**), we showed that NMDAR antagonism, TrkB agonism, and opioid receptor agonism in the basal ganglia and M1 contribute to the therapeutic effects in LID. According to the *cortical disinhibition hypothesis*, ketamine causes a burst of glutamate due to reduced cortical gamma-aminobutyric acid release from PV interneurons in M1. As mentioned previously, ketamine antagonizes NMDARs expressed on PV neurons, presumably decreasing PV-neuron activity. The resulting decrease disinhibits target glutamatergic M1 principal cells, leading to glutamate and BDNF release onto downstream striatal medium spiny neurons, activation of α-amino-3-hydroxy-5-methyl-4-isoxazolepropionic acid receptors, depolarization, and to stimulation of TrkB, activating mammalian target of rapamycin complex 1 and extracellular signal-regulated kinase 1/2. Ketamine also binds to TrkB receptors and leads to increased membrane availability and activation, enhancing BDNF signaling further, an interaction that can be inhibited by pravastatin (Bartlett et al., 2025), which blocks both the long-term anti-depressive and anti-LID activities of ketamine. The activation of the BDNF-signaling pathway, a known master regulator of neuroplasticity, leads to a reduction of increased mushroom spines in the medium spiny neurons in the dyskinetic striatum (Bartlett et al., 2020). In addition, ketamine is a positive allosteric modulator for opioid receptors known to activate extracellular signal-regulated kinase 1/2, leading to the acute anti-parkinsonian effects, likely driven by increased delta- and mu-opioid receptor activation.

While there is evidence for the *cortical disinhibition hypothesis*, its predictions do not entirely conform to observations in Vishwanath et al. (2024), where ketamine did not increase the mean firing rates in M1 during LID, but, instead, reorganized interactions between neurons. This experiment, however, did not separate the activities of PV neurons from other cell types. Future experiments involving optical tagging or targeted manipulation of PV-expressing neurons during LID and ketamine treatment would help resolve this issue.

**Conclusion and clinical relevance:** The novel neural ensemble state produced by ketamine in M1 combined with ketamine-induced BDNF signaling may contribute to acute and long-lasting reductions in LID. This suggests that ketamine works as a “*chemical deep brain stimulation*” by disrupting neuron-neuron interactions involved in aberrant hypersynchrony in LID and restoring cortical control over downstream motor centers. We have reported retrospective PD cases that benefitted from sub-anesthetic ketamine infusions, and we conducted a Phase 1 clinical trial that has shown safety, tolerability, and signs of efficacy, with a reduction of LID from baseline up to 3 months post treatment (NCT06021756). Ketamine did not worsen the PD symptoms or block the beneficial effects of levodopa; rather, ketamine had a motor benefit. A multicenter placebo-controlled double-blind Phase 2 clinical trial is in the planning stages. These clinical results add further significance to our preclinical mechanistic investigations and support this repurposing approach to use low-dose ketamine infusion as a treatment for individuals with LID.

*We thank Drs. Andy Fuglevand, Asier Aristieta, and Abhilasha Vishwanath (The University of Arizona, USA) for their helpful comments.*

*This work was supported by Arizona Biomedical Research Commission [ADHS18-198846], the National Institute of Health NINDS [R56-NS109608 and R01-NS122805], and Davies, Robert and Peyton, Parkinson’s Disease Research Fund to TF.*

*Torsten Falk has a patent for the use of ketamine as a novel treatment for dopamine precursor levodopa-induced dyskinesia associated with PD that was licensed to PharmaTher Inc. in 2020. He consulted for PharmaTher Inc. in 2021 and 2023 and received travel support in 2022 and 2023.*

**Additional file:** *Open peer review report 1.*

OPEN PEER REVIEW REPORT 1
