# Supplementary material for: Motor cortex in levodopa-induced dyskinesia: Systems and molecular changes after sub-anesthetic ketamine treatment
Source: Neural Regen Res. 2025 Sep 29;21(8):3533–4. doi: 10.4103/NRR.NRR-D-25-00176 (PMC13452678; doi:10.4103/NRR.NRR-D-25-00176)
Supplement: Supplementary file 1 [file NRR-21-3533_Suppl1.pdf]

OPEN PEER REVIEW REPORT 1

Name of journal: Neural Regeneration Research
Manuscript NO: NRR-D-25-00176
Title: Motor cortex in levodopa-induced dyskinesia: Systems and molecular changes after sub-anesthetic ketamine treatment
Reviewer's Name: Uwe Rudolph
Reviewer's country: USA

COMMENTS TO AUTHORS

This Perspective article discusses how sub-anesthetic ketamine treatment on levodopa-induced dyskinesia (LID) exerts an ani-dyskinetic effect. In line with the character of the Perspective series, it appears to focus on results that the same group published recently in the journal Brain (A Vishwanath et al., 2024). The figures in the Perspectives article are not contained in the Brain article and summarize the findings very nicely, almost in the style of a graphical abstract. A major point that the authors are making is that ketamine reorganized the pattern of M1 neuron-pair correlations (Figure 1) and at the molecular level activates BDNF-dependent signaling pathway both via NMDA receptor antagonism at PV interneurons and some activating effects on TrkB and the mu and delta opioid receptors on medium spiny neurons. The also mention the limitation that recent experiments were unable to separate between ketamine's effects on these cell types. Ketamine did not increase the mean firing rates at M1 during LID, which would have been predicted by the cortical disinhibition hypothesis. The article is well-written and informative and thus recommended for publication.

Minor point
Line 6: "GluN2b" should be "GLuN2B".
